# Flexural Strength of Preheated Resin Composites and Bonding Properties to Glass-Ceramic and Dentin

**DOI:** 10.3390/ma9020083

**Published:** 2016-01-29

**Authors:** Matthias Richard Kramer, Daniel Edelhoff, Bogna Stawarczyk

**Affiliations:** Department of Prosthodontics, Dental School, Ludwig-Maximilians-University Munich, Goethestrasse 70, 80336 Munich, Germany; Matthias_Richard.Kramer@med.uni-muenchen.de (M.R.K.); Daniel.edelhoff@med.uni-muenchen.de (D.E.)

**Keywords:** dental composite, preheating, flexural strength, shear bond strength, interfacial tension

## Abstract

To test the impact of preheating (25, 37, 54, or 68 °C) of TetricEvoCeram (TEC), FiltekSupremeXT (FSXT), and Venus (V) on flexural strength (FS), shear bond strength (SBS) and interfacial tension (IFT). FS was tested with TEC and FSXT. For SBS, glass-ceramic and human dentin substrate were fabricated and luted with the preheated resin composite (RC). SBSs of 1500 thermal cycled specimens were measured. For IFT, glass slides covered with the non-polymerized RC were prepared and contact angles were measured. Data were analyzed using 2/1-way *ANOVA* with Scheffé-test, and *t*-test (*p* < 0.05). Preheated TEC (37–68 °C) showed higher FS compared to the control-group (25 °C) (*p* < 0.001). FSXT presented higher FS than TEC (*p* < 0.001). For SBS to dentin higher values for FSXT than TEC were found. The preheating temperature showed no impact on SBS to dentin. SBS to glass-ceramic revealed a positive influence of temperature for TEC 25–68 °C (*p* = 0.015). TEC showed higher values than V and FSXT (*p* < 0.001). IFT values increased with the preheating temperature. A significant difference could be observed in every RC group between 25 and 68 °C (*p* < 0.001).

## 1. Introduction

Dental resin composites (RC) represent one of the most frequently used materials in dentistry in the field of tooth colored restorations in anterior and posterior dentition as well as adhesive cementation. They have become an important part of modern minimum invasive treatment concepts. However, some specific properties like microleakage [1], deterioration [2] tendency to plaque retention and discoloration, insufficient hardness and wear resistance, flexural strength or flexural modulus [3] and sensitivity towards water/saliva [4,5] have been discussed. Driven by some negative long-term clinical results and the immense competition on the dental market, in the last decade important improvements of the mechanical properties of RCs were conducted, predominantly by the increase of the anorganic filler part. Increasing filler parts cause in general a higher RC-viscosity, which could reduce the ease of clinical application. Therefore, options to improve the rheological behavior of high filled RCs by using ultrasonic treatment [6] or preheating [7,8,9] are of primary interest for the clinician. Special small size chairside furnaces are commercially available to facilitate the chairside use of preheated RCs. Studies investigating the flowability of preheated nanohybrid RCs showed a decrease in film thickness and viscosity by 25%. An even larger decrease could be observed with microhybrid RCs (minus 70%) [7,8,9]. This fact enables the use of RCs with high organic filler content for the adhesive cementation of indirect manufactured inlays, onlays, or facial veneers. Beside several benefits in clinical application, this technique leads to important mechanical improvements: First of all to a higher fracture resistance of adhesive cementation [10] and secondly to the improvement of the mechanical properties as a result of higher filler contents [11,12]. Additional benefits of this technique have been seen in a higher curing efficiency since the degree of conversion rises [13,14], whereas the needed light intensity declines. For example it could be shown in two *in vitro* studies, that at 40 °C a 50% shorter curing time leads to the same degree of conversion [15,16]. On the other hand, the same curing time results in less residual monomer, minimizing the risk of hypoallergenic reactions [17,18,19,20]. Another study reported less leakage in class II restorations by using a preheated RC [21]. A better adaptation to the tooth was given as an explanation. One control group had a flowable liner at the bottom of the cavity, which still scored inferior to the preheated group.

To the best knowledge of the authors, only insufficient data exist on the mechanical properties of preheated RCs. Flexural strength (FS) provides a reliable predictor for clinical durability, for example for the risk of fracture of anterior and posterior restorations a significant correlation was reported [22]. Similarly, knowledge of the adhesion to glass-ceramic and tooth structure is very scarce. The bond to these two surfaces is important for the adhesive cementation of most indirect restorations. The bonding properties can be measured using the tensile or shear bond strength (SBS) test. Interfacial tension (IFT) evaluated by contact angle measurements provides additional information about the wettability [23].

This study investigated the mechanical and bonding properties of preheated RCs. The hypothesis investigated was that preheating of RCs shows no negative impact on flexural strength and bonding properties to glass-ceramic or dentin.

## 2. Materials and Methods

**Figure 1:**
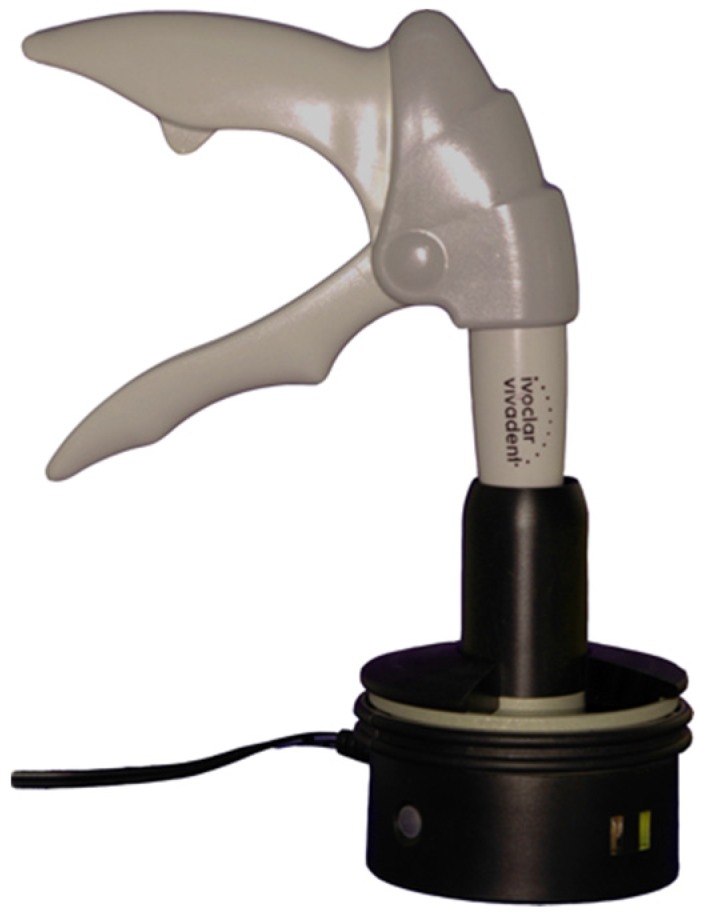
Calset applicator.

Three selected restorative nanohybrid RCs: Tetric EvoCeram (TEC) (Ivoclar Vivadent, Schaan, Liechtenstein), Filtek Supreme XT (FSXT) (3M ESPE, Seefeld, Germany), and Venus (V) (Heraeus Kulzer, Hanau, Germany) were tested in this study (Table 1). The influence of preheating on these RCs was determined using flexural strength (FS), shear bond strength (SBS), and interfacial tension (IFT) measurements. Bonding to human dentin was tested with TEC and FSXT and to glass-ceramic with TEC, FSXT, and V. The RCs were preheated in a commercial device (Calset, AdDent, Danbury, CT, USA) at the following temperature levels: room temperature (25 °C; not preheated, acted as control group), 37 °C, 54 °C, and 68 °C (Figure 1)*.*

**Table 1:** Designation of the resin composites (RCs) used in the present study. Showing composition, filler size, and filler content according to manufacturer instruction as well as used lot number (lot no.).

Material	Composition	Filler Size	Filler Content (wt%/vol%)	Lot no	Manufacturer
*TEC*	Resin: Bis-GMA, UDMA, TEGDMA Filler: barium glass, ytterbium trifluoride, mixed oxide	0.04–3 µm (mean: 0.55 µm)	76/55	S09551	Ivoclar Vivadent (Schaan, Liechtenstein)
*FSXT*	Resin: Bis-GMA, Bis-EMA, UDMA, TEGDMA Filler:zirconia, silica	5–20 nm with mean clusters of 0.6–1.4 µm	79/60	8RG	3M ESPE (Seefeld, Germany)
*V*	Resin: Bis-GMA, TEGDMA Filler: barium Aluminium fluoride glass, silicon dioxide	0.04–2 µm	78/61	010405	Heraeus Kulzer (Hanau, Germany)

Bis-GMA: Bis-phenol-A-diglycidylmethacrylate; TEGDMA:Triethylene-glycol-dimethacrylate; UDMA: Urethane-dimethacrylate; Bis-EMA: ethoxylated Bis-phenol-A-diglycidyldimethacrylate.

### 2.1. Three-Point Flexural Strength Measurements

Eighty bar-shaped specimens (2 × 2 × 25 mm^3^), 40 per RC material, were fabricated in a half-split stainless steel mold (ISO 4049) [24]. Preheated RC was injected in a single increment into the mold covered with a polyester strip and a glass plate from both sides. The specimens were irradiated at the top and bottom surfaces in five serial exposures of 20 s, overlapping the previously irradiated section with half the diameter of the light guide (Elipar S10, 3M ESPE, Seefeld, Germany). The intensity of the LED light-curing unit was measured (1200 mW/cm^2^) using an analyzing device (Marc V3; BlueLight Analytics Inc., Halifax, NS, Canada). Thereafter, the specimens were polished with silicone carbide (SiC) paper up to grit size P2400. The dimensions were measured with a digital caliper (CAPA 150; Tesa SA, Renens, Switzerland) with 0.01 mm accuracy.

Before testing, the specimens were stored in distilled water at 37 °C for 24 h (HERA cell 150 Thermo scientific, Heraeus Kulzer, Hanau, Germany). Thereafter, the specimens were subjected to thermocycling (Thermocycler THE 1100, SD Mechatronik, Feldkirchen-Westerham, Germany) for 1500 cycles between 5 and 55 °C with a dwelling time of 20 s and transfer time of 10 s.

**Figure 2:**
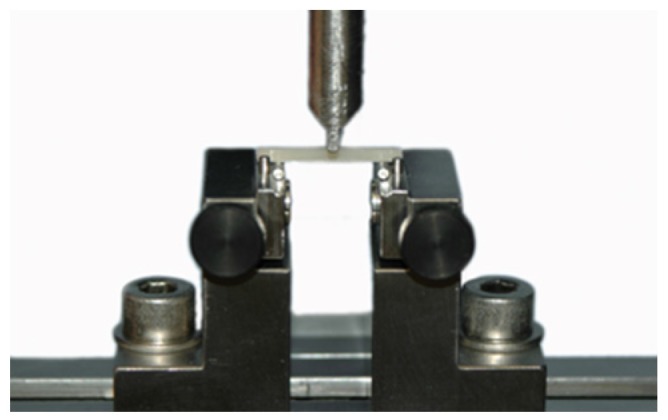
Three-point flexural strength test.

The three-point bending test was carried out in a Universal Testing Machine (Zwick 1445, Zwick, Ulm, Germany) at a crosshead speed of 1 mm/min (Figure 2). 

Specimens were placed on two parallel supports separated by 20 mm, and loaded until fracture. Supports and loading piston were steel knife edges rounded to a radius of 0.8 mm. Flexural strength (σ) values, in MPa, were calculated using the following formula [24]:

(1) $$\sigma =\frac{\;3Fl}{2bh²}$$

*F*: load at fracture (N)
*l*: distance between the supports (mm)
*b*: width of the specimen (mm)
*h*: height of the specimen (mm)

### 2.2. Shear Bond Strength Measurements 

SBS measurements were performed with human dentin and glass-ceramic specimens.

At first human dentin specimens were prepared from 80 extracted caries-free human third molars stored in 0.5% *chloramine T* (Lot no: 53110, CAS: 7080-50-4, Sigma-Aldrich Laborchemikalien GmbH, Seelze, Germany) at room temperature (25 °C) for a maximum of 7 days after extraction followed by storage in distilled water at 5 °C for a maximum of 6 months until use [25]. The teeth were embedded in auto-polymerizing acrylic resin (ScandiQuick, Scan-Dia, Hagen, Germany) with the buccal surface facing upwards. The buccal surfaces of the embedded teeth were ground flat in a polishing machine (LaboPol-21; Struers, Ballerup, Denmark) under water cooling with a series of SiC-papers up to P500 grit until a dentin area of at least 4 × 4 mm^2^ was exposed. Dentin was etched for 15 s with 35% phosphoric acid (Ultra-Etch; Ultradent Products, South Jordan, UT, USA, lot no.: B5MKW), rinsed with water for 30 s and dried carefully leaving the dentin surface with a slightly glossy wet appearance. A maleic acid-containing primer (Syntac Primer; Ivoclar Vivadent, lot no.: N31553) was applied for 15 s and gently air-dried before application of a second primer (Syntac Adhesive; Ivoclar Vivadent, lot no.: N35661) for 10 s, again followed by gentle air-drying. 

For fabrication of the glass-ceramic SBS specimens, leucite glass-ceramic blanks (IPS Empress CAD, Ivoclar Vivadent, lot no.: R42565) were cut (Secutom-50, Struers) in slices of 3 mm thickness and embedded in epoxy resin (Specific Resin 20, Struers). Subsequently, all substrate specimens were polished (Tegra Force/Tegra Pol, Struers) with diamond discs (MD-Piano, Struers) P220 up to P1200. Before initiating the bonding procedure, the specimens were cleaned for 10 min in an ultrasonic bath in distilled water (Bransonic Ultrasonic Cleaner 3510 E-DTH, Branson, Danbury, CT, USA), air-dried and randomly divided into three RC groups (*n* = 40).

The glass-ceramic surfaces were etched with 9.5% hydrofluoric acid gel (Porcelain Etch, Ultradent, South Jordan, UT, USA, lot no.: B6X7B) for 60 s. The etched substrates were washed and rinsed thoroughly to remove the residual acid, air-dried, and coated with a 3-methacryloxypropyltrimethoxy (MDP) silane coupling agent (Monobond Plus, Ivoclar Vivadent, lot no.: R26662) and allowed to air dry for an additional 5 min. 

Onto both conditioned surfaces (dentin/glass-ceramic) an acrylic hollow cylinder (D+R Tec, Birmensdorf, Switzerland) with an inner diameter of 2.9 mm was pressed using a special bonding device described in detail previously [26]. A bonding agent (Heliobond; Ivoclar Vivadent, lot no.: L43615/ P25961) was applied with a microbrush, thinned with mild air, and after carefully removing all excess material the bonding agent was light-cured (Elipar S10) for 10 s. Thereafter, the RC material was filled in a 2 mm increment into the opening of the cylinder at the given temperature (25 °C, 37 °C, 54 °C, 68 °C) (*n* = 10 per RC and preheating level), and light-cured for 40 s (Elipar S10). Consequently, the luted specimens were aged as described in FS tests.

**Figure 3:**
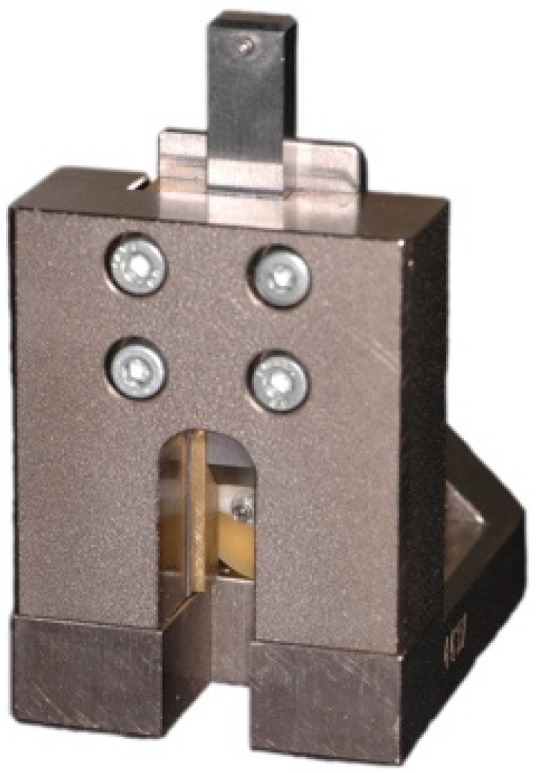
Shear bond strength test.

A Universal Testing Machine (Zwick 1445, Zwick, Ulm, Germany) was used for SBS measurements. The specimens were positioned in the sample holder of the testing machine with the dentin surface parallel to the chisel-shaped loading piston (Figure 3). Shear loading was applied to the adhesive interface at a crosshead speed of 1 mm/min until debonding occurred. Load at debonding was recorded and SBS (σ) values, in MPa, were calculated according to the equation:

(2) $$\sigma \;=\;\frac{F}{A}$$

*F*: load at fracture (N)
*A*: adhesive area (mm^2^)

For failure type analysis, the debonded area was analyzed with a stereomicroscope (Stemi DV 4, Carl Zeiss MicroImaging GmbH, Göttingen, Germany) at 20× magnification. Failure of each specimen was classified into one of the four categories: (i) adhesive: fracture occurred at the dentin/glass-ceramic and RC interface, (ii) cohesive in RC: fracture occurred in RC, (iii) cohesive in dentin/glass-ceramic: fracture occurred in substrate, (iv) mixed: combination between adhesive and cohesive. One calibrated examiner, who was unaware of the group allocation, evaluated all failure types.

### 2.3. Interfacial Tension Measurements

The interfacial tension (IFT) of the unpolymerized RC surfaces in all temperature levels was calculated using contact angle performed with water (polar liquid) and di-iodomethane (CAS: 15.842-9, Sigma-Aldrich, lot no.: S65447–448) (disperse liquid) parameters. The RCs were preheated to the given temperature within 3 min and then spread thinly on a glass slide (Menzel, Braunschweig, Germany) using a mixing spatula producing a film thickness of about 0.2 mm.

The whole procedure happened under filtered light (OG530, Schott, Mainz, Germany) to maintain a wavelength over 500 nanometers to avoid a curing of the RC. A long pass filter ensures a transmittance τ of less than 1 × 10^−5^ under 500 nanometers. Afterwards, the glass slides were placed onto the heating device (Calset, AdDent, Danbury, CT, USA) to keep the temperature throughout the whole measuring period. In addition, the temperature was controlled with an infra-red thermometer (IR 900-30S, Voltcraft, Conrad Electronic SE, Hirschau, Germany). Onto every specimen three drops of distilled water and three drops of di-iodomethane were placed. Since the distance of the drop to the camera stayed the same, the diameter could be measured with a virtual scale and kept constant to maintain an equal volume of water and di-iodomethane.

The contact angle was measured with the sessile drop method. In a special device (Easydrop DSA20E, Krüss, Hamburg, Germany) a photo was taken with a digital camera (IEEE1394b–Stingray F-046, Allied Vision Technologies GmbH, Stadtroda, Germany) using backlighting. In order to standardize the measurements, the water drop was captured after 10 s and the di-iodomethane drop after 30 s. The specimen was positioned parallel to the light beam to get the silhouette of the drop. Di-iodomethan produced a flat drop because of the wide spreading. To eliminate the light diffusion of the specimen/composite itself a light shade was used. A computer program (DSA 4 Software, Krüss, Hamburg, Germany) detected baseline, outline, and the contact angle. The software calculated the interfacial tension (IFT) values of the polar und the disperse part using the method of Owens, Wendt, Rabel, and Kaelble [27,28]. Two equations provide the basis for this way to get the IFT. The first one is the equation by *Young*, giving the correlation between the contact angle and the IFT of the solid specimen [27]:

(3) $$\text{cos}\theta =\frac{IFT_{s}-IFT_{ls}}{IFT_{l}}$$

$IFT_{s}=interfacial\;tension\;solid\;\left(mN/m\right)$
$IFT_{l}=interfacial\;tension\;liquid\;\left(mN/m\right)$
$IFT_{ls}=interfacial\;energy\;liquid\;to\;solid\;\left(mN/m\right)$
θ=contact angle (degree)

The second one is the geometric mean by Owens, Wendt, Rabel, and Kaelble.

(4) $$IFT_{sl}=IFT_{s}+IFT_{l}-2*\left(\sqrt{IFT_{s}^{d}IFT_{l}^{d}}+\sqrt{IFT_{s}^{p}IFT_{l}^{p}}\right)$$

$IF{T_{s}}^{d}=disperse\;part\;of\;interfacial\;tension\;\left(mN/m\right)$
$IF{T_{s}}^{p}=polar\;part\;of\;interfacial\;tension\;\left(mN/m\right)$
$IFT_{sl}=interfacial\;energy/tension\;solid\;to\;liquid\;\left(mN/m\right)$

Putting those two together, it is possible to get an even function:

(5) $$\underset{y}{\underset{︸}{\frac{IFT_{l}*\left(\text{cos}\theta +1\right)}{2*\sqrt{IFT_{l}^{d}}}}}=\underset{m}{\underset{︸}{\sqrt{IFT_{s}^{p}}}}\underset{x}{\underset{︸}{*\frac{\sqrt{IFT_{l}^{p}}}{\sqrt{IFT_{l}^{d}}}}}+\underset{b}{\underset{︸}{\sqrt{IFT_{s}^{d}}}}$$

Calculating two points of the straight line leads us to the gradient and the y-intercept respectively the polar and disperse part of the solid specimen.

**Figure 4:**
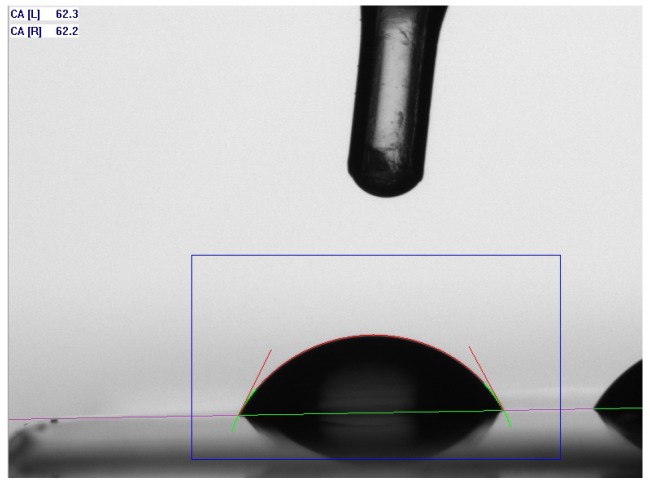
Contact angle measurements.

### 2.4. Statistical Analysis

The Statistical Package for the Social Science Version 20 (*SPSS*, Chicago, IL, USA) was used to calculate descriptive statistics (mean, standard deviation (SD), 95% confidence interval (95% CI)) of all measured results. Approximate normality of data distribution was tested with the Kolmogorov-Smirnov and Shapiro-Wilk tests. Furthermore, the Weibull modulus and characteristic strength were calculated. Two-way and one-way ANOVA followed by Scheffé’s *post-hoc* test, as well as unpaired two-sample *t*-test were conducted. For flexural strength data non-parametric tests, such as Kruskal-Wallis and Mann-Whitney were used. Relative frequencies of the different fracture types together with the 95% CI (using Ciba-Geigy tables) were provided. All results of the statistical analyses with *p*-values smaller than 0.05 were considered to be statistically significant. 

## 3. Results

According to the Kolmogorov-Smirnov and Shapiro-Wilk test, one (12.5%) of eight flexural strength groups showed significant deviation of normal distribution (37 °C, TEC). The remaining groups (87.5%) were normally distributed. Therefore, for flexural strength tests non-parametric analysis was used. The SBS and IFT values were normally distributed and parametric tests were performed.

### 3.1. Three-Point Flexural Strength

#### 3.1.1. Impact of Temperature for Each RC

TEC showed significantly higher flexural strength values after preheating (37–68 °C) compared to non-preheated control group (25 °C) (*p* < 0.001). In contrast, FSXT presented no impact of preheating on the flexural strength results (*p* = 0.372) (Table 2).

**Table 2:** Mean with standard deviation for flexural strength (FS) (MPa) appendant Weibull modulus and characteristic FS as well as 95% confidence interval (CI) for each tested RC by each pre-heating temperature.

RC	Pre-Heating	Mean ± SD	95% CI	Weibull Modulus	95% CI	Characteristic FS	95% CI
**FSXT**	25 °C	92 ± 5 ^a/B^	(87; 96)	21	(9; 40)	94	(90; 98)
	37 °C	91 ± 7 ^a/B^	(85; 97)	14	(6; 27)	95	(89; 100)
	54 °C	96 ± 4 ^a/B^	(91; 99)	28	(13; 53)	98	(94; 100)
	68 °C	94 ± 4 ^a/B^	(89;97)	26	(12; 51)	96	(92; 99)
**TEC**	25 °C	63 ± 7 ^a/A^	(56; 68)	10	(4; 20)	66	(60; 70)
	37 °C	73 ± 5 ^b/A^	(68; 77)	21	(9; 40)	75	(71; 78)
	54 °C	79 ± 6 ^b/A^	(73; 84)	15	(6; 29)	82	(77; 86)
	68 °C	76 ± 8 ^b/A^	(69; 83)	12	(5; 23)	79	(73; 84)

^a,b^ Different lower case superscript presents significant difference between the results of each temperature level within one RC group; ^A,B^ Different upper case superscript presents significant difference between the results of each RC within one temperature level.

#### 3.1.2. Impact of RC for Each Temperature

In general, higher flexural strength results for FSXT than for TEC were observed, regardless of the experimental temperature (*p* < 0.001).

#### 3.1.3. Weibull Modulus

The Weibull analysis with respect to Weibull modulus generated values between 10 (TEC, 25 °C) and 28 (FSXT, 54 °C) nevertheless without significant differences. 

### 3.2. Shear Bond Strength to Dentin

According to two-way *ANOVA*, RC materials showed an impact (*p* < 0.001) on the SBS results. However, the experimental temperature presented no statistical impact (*p* = 0.402) and the interaction between the both parameters (*p* = 0.448) was not significant.

#### 3.2.1. Impact of RC for Each Temperature

FSXT presented in all experimental temperature groups higher SBS to dentin than the RC TEC (25 °C: *p* = 0.002; 37 °C: *p* = 0.005; 54 °C: *p* < 001; 68 °C: *p* = 0.008) (Table 3).

**Table 3:** Mean with standard deviation for shear bond strength (SBS) to dentin (MPa) appendant Weibull modulus and characteristic SBS as well as 95% confidence interval (CI) for each tested RC by each pre-heating temperature.

RC	Pre-Heating	Mean ± SD	95% CI	Weibull-Modulus	95% CI	Characteristic SBS	95% CI
**FSXT**	25 °C	12 ± 3.5 ^a/B^	(8; 15)	3.3	(0; 7)	13	(9; 16)
	37 °C	14 ± 5.0 ^a/B^	(9; 18)	3.1	(0; 6)	16	(11; 20)
	54 °C	15 ± 5.1 ^a/B^	(10; 19)	3.8	(0; 8)	17	(12; 20)
	68 °C	13 ± 4.3 ^a/B^	(8; 17)	3.8	(0; 8)	14	(10; 17)
**TEC**	25 °C	7.1 ± 1.3 ^a/A^	(5; 9)	6	(2; 12)	8	(5; 9)
	37 °C	8.3 ± 3.0 ^a/A^	(5; 11)	2.5	(0; 5)	9	(6; 13)
	54 °C	7 ± 1.8 ^a/A^	(4; 9)	4.5	(1; 9)	8	(5; 9)
	68 °C	7.9 ± 3.2 ^a/A^	(4; 11)	3.4	(0; 7)	9	(6; 11)

^a,b^ Different lower case superscript presents significant difference between the results of each temperature level within one RC group; ^A,B^ Different upper case superscript presents significant difference between the results of each RC within one temperature level.

#### 3.2.2. Weibull Modulus

Weibull analyses with respect to Weibull modulus were performed and resulted in no significant difference between the groups in a range between 2.5 (TEC, 37 °C) and 6.0 (TEC).

#### 3.2.3. Fracture Type Analysis

In all groups adhesive failure types were predominant. No cohesive failures in RC were observed. Cohesive fracture modes in dentin occurred once.

### 3.3. Shear Bond Strength to Ceramic

The two-way *ANOVA* showed significant interaction between RC and temperature (*p* < 0.001). Consequently, several different analyses were computed and split at levels of RC material and temperature depending on the hypothesis of this study. The descriptive statistics are presented in Table 4.

**Table 4:** Mean with standard deviation for SBS to ceramic (MPa) appendant Weibull modulus and characteristic SBS as well as 95% confidence interval (CI) for each tested RC by each pre-heating temperature.

RC	Pre-Heating	Mean ± SD	95% CI	Weibull-Modulus	95% CI	Characteristic SBS	95% CI
**V**	25 °C	20 ± 5 ^a/A^	(15; 24)	5.2	(1; 11)	21	(18; 35)
	37 °C	14 ± 5 ^a/A^	(9; 18)	3	(0; 6)	16	(11; 20)
	54 °C	16 ± 4 ^a/A^	(12; 20)	4.4	(1; 9)	18	(14; 21)
	68 °C	15 ± 6 ^a/A^	(10; 20)	3.9	(1; 8)	17	(13; 20)
**FSXT**	25 °C	23 ± 5 ^a/A^	(17; 27)	5.1	(1; 10)	24	(20; 28)
	37 °C	19 ± 6 ^a/A^	(14; 24)	3.8	(1; 8)	21	(16; 26)
	54 °C	17 ± 8 ^a/A^	(10; 23)	2.3	(0; 5)	19	(13; 26)
	68 °C	18 ± 6 ^a/A^	(12; 23)	3.9	(1; 8)	19	(15; 24)
**TEC**	25 °C	25 ± 7 ^a/A^	(19; 30)	4	(1; 8)	27	(22; 33)
	37 °C	34 ± 9 ^a,b/B^	(27; 41)	5	(1; 10)	37	(31; 43)
	54 °C	38 ± 16 ^a,b/B^	(25; 51)	2.7	(0; 6)	43	(32; 56)
	68 °C	44 ± 15 ^b/B^	(31; 55)	3.4	(0; 7)	48	(38; 59)

^a,b^ Different lower case superscript presents significant difference between the results of each temperature level within one RC group; ^A,B^ Different upper case superscript presents significant difference between the results of each RC within one temperature level

#### 3.3.1. Impact of Temperature for Each RC

The influence of the preheating temperature in the RC groups only showed a significant difference within the TEC group between 25 °C and 68 °C (*p* = 0.015).

#### 3.3.2. Impact of RC for Each Temperature

The one-way *ANOVA* was calculated, resulting in no significant differences at temperature 25 °C, but in significantly higher SBS values at temperature 37 °C, 54 °C, and 68 °C for TEC compared to V and FSXT (*p* < 0.001, respectively).

#### 3.3.3. Weibull Modulus

The Weibull modulus was calculated for every group (*n* = 10) ranging from 2.3 (FSXT, 54 °C) to 5.2 (V, 25 °C), however no statistical differences between tested groups were observed.

#### 3.3.4. Fracture Type Analysis

Except one specimen, all fracture types were cohesive in glass-ceramic.

### 3.4. Interfacial Tension

The two-way *ANOVA* described an interaction between temperature and RC for IFT (*p* < 0.001). Several different analyses were computed and split at levels of RC materials and temperature depending on the hypothesis of this study.

#### 3.4.1. Impact of Temperature for Each Resin Composite (*p* < 0.001)

V showed significant higher values for 68 °C and for 54 °C only compared to 25 °C. FSXT provides higher values for 54 °C and 68 °C *versus* 25 °C and 37 °C. Within TEC there was no significant difference between 25 °C and 37 °C but higher values for 54 °C were followed by even higher at 68 °C (Table 5).

**Table 5:** Mean with standard deviation for interfacial tension (IFT) (mN/m) as well as 95% confidence interval (CI) for each tested RC by each pre-heating temperature.

RC	Pre-Heating	Mean ± SD	95% CI
**V**	25 °C	59 ± 0.4 ^a/A^	(57; 60)
	37 °C	59 ± 0.9 ^a,b/A^	(57; 60)
	54 °C	60 ± 0.8 ^b/A^	(58; 61)
	68 °C	63 ± 1 ^c/A^	(61; 64)
**FSXT**	25 °C	49 ± 0.6 ^a/C^	(47; 50)
	37 °C	50 ± 0.6 ^a/C^	(48; 51)
	54 °C	53 ± 1 ^b/C^	(50; 54)
	68 °C	54 ± 0.9 ^b/C^	(51; 55)
**TEC**	25 °C	53 ± 0.5 ^a/B^	(51; 54)
	37 °C	54 ± 0.7 ^a/B^	(51; 54)
	54 °C	55 ± 0.6 ^b/B^	(53; 55)
	68 °C	56 ± 0.6 ^c/B^	(54; 57)

^a,b,c^ Different lower case superscript presents significant difference between the results of each temperature level within one RC group; ^A,B,C^ Different upper case superscript presents significant difference between the results of each RC within one temperature level.

#### 3.4.2. Impact of Resin Composite for Each Temperature (*p* < 0.001)

Regarding the temperature levels every resin composite showed a significant influence: Highest values resulted for V followed by TEC and FSXT.

## 4. Discussion

Given by the temporary increased flowability, preheating of higher filled RCs can facilitate the clinical application [7,8] in both, direct composite layering and increment technique, as well as adhesive cementation of indirect restorations. On the other hand this technique may affect the mechanical properties of RCs. The aim of this study was to verify that the use of preheated light curing RCs for the adhesive cementation of indirect glass-ceramic restorations has no negative impact on the mechanical properties and bonding characteristics.

The bonding interface can be separated into three different layers: dentin, *RC*, glass-ceramic. In the first step, the RC itself was tested. Therefore three-point flexural strength tests were performed. The first hypothesis, that the preheating of the RC does not decrease the flexural strength, could be accepted. TEC showed higher results for the preheated RCs than for the control-group. It can be assumed that the higher degree of conversion of preheated RCs has a positive influence on the flexural strength [13,14,29]. In general ISO 4049:2000 norm suggests, that minimal flexural strength values for so called Type 1 RCs should be 80 MPa. FSXT fulfilled this standard thoroughly for every tested temperature. TEC showed the highest flexural strength values (79 MPa) for the 54 °C preheated RC group. The additional thermocycling might be a reason for the lower values of TEC. Another study also reported lower flexural strength values for TEC (79 MPa) after three days of water storage (37 °C) than after one day storage (99 MPa) [30]. Other investigations could confirm these findings for flexural strength [30,31]. 

In addition to the study of mechanical properties of the adhesive, respectively the RC itself, the contact area between RC and dentin was examined. Macro SBS tests verified the hypothesis that the SBS does not decrease with a higher temperature of the RC. FSXT showed higher results in all temperature levels than TEC. Another study did not find significant differences between those two RCs. Though the values were higher than the ones in the current study (19.2 ± 4.4 MPa to 29.8 ± 5.7 MPa [32]). Comparing the two studies several differences could be observed: the adhesive system, the duration of water storage, thermocycling and the adhesive area. A more recent study found very similar results to the present study for FSXT (in a range from 11.01 ± 4.56 MPa to 15.36 ± 6.00 MPa) [33], although differing in a few parameters, like other adhesives and no thermocycling. A review [34] demonstrated the diversity of the results of SBS tests, ranging from 3–61 MPa. A classification into adhesive systems still yielded differences of at least 16 MPa. The lack of standardization is one of the problems with SBS tests. Simple testing parameters, like crosshead speed [35,36], aging process [33,37] or storage conditions of the natural teeth [38,39,40], have demonstrable influence on the results. Another deficit emphasized by Scherrer [34] was the fracture pattern. As far as possible, in the present study, by light microscopy, fracture patterns on the dentin surface were rated to be mainly adhesive. This reinforces the fact that dentin bonding still seems to be a critical issue, where further developments will be needed.

In contrast the bonding between RC and glass-ceramic can only be separated through high forces. The SBS test and the work of adhesion values did not show a decrease due to preheating. In this regard the hypothesis could be accepted. Higher temperature even showed a positive impact on the work of adhesion and on one group of SBS tests, resulting in higher values for preheated RCs. A comparison with other studies is quite difficult due to the variety of used components. No study was found reflecting the same set up in SBS test or matching all components used. At least different studies about SBS tests with leucite-reinforced glass-ceramics are available. One study [41] used a CAD/CAM-glass-ceramic pretreated with Monobond Plus (CM, Ivoclar Vivadent, Schaan, Liechtenstein), a silane containing coupling agent. SBS values were at 26.3 ± 7.5 MPa and from 29.1 ± 12.5 MPa to 47.4 ± 8.6 MPa for other silane coupling agents, being very close to the TEC group [41]. Other studies [42,43,44] described values from 11.8 MPa to 24.6 MPa. All of them applied hydrofluoric acid to the glass-ceramic to produce the micro retentive pattern. The range of the test results correlates well with the V and FSXT groups. Nevertheless many parameters like surface treatment, polishing or air-abrading before etching, crosshead speed or storage conditions were not consistent. Besides all the differences, in one point a high consensus was found: The fracture pattern plays an important role [45,46]. Almost every specimen showed a similar pattern with a cohesive part in the glass-ceramic. Several studies [41,42,43] also observed predominately cohesive fractures. One [43] even defined a threshold at about 17 MPa where “the mode of fracture was primarily cohesive within the porcelain” [43]. This result supports the present observations. However, another study using FEA analyses showed the stress and tension areas occurring in a SBS test [47]. As a result of the uneven distribution of the forces and the specific properties of ceramics (brittle material), fragments of variable size break out from the specimens. On the one hand this leads to a relatively high standard deviation [34]. On the other hand it is impossible to achieve a quantitative evaluation of the bonding between the ceramic and the RC, because a good deal of the calculated SBS only reflects cohesive forces in the ceramic. All in all, the tests demonstrated that the bond is stronger than the inherent strength of the restorative material, which thus is not the weak point at the adhesive interface.

To support the results of the destructive method of SBS test, the non-destructive method of contact angle measurements was used. The IFT values did not decrease and also a slight increase could be observed, which was partly significant. Because of higher wettability in both the disperse and the polar part, the values of the preheated RC increased. Several potential reasons could be taken into consideration: A change in surface structure suggests itself. Experiments showed that, for example, shorter micropili on a surface provide better wetting characteristics and probably a change from *Cassie* wetting state to a *Wenzel* state [48]. Another influence is given by the *Eötvös*-Rule: the surface tension of a liquid is indirectly proportional to its temperature. A lower surface tension leads thereby to a lower contact angle [46]. Improved adaptation after preheating has also been suggested by others e.g., as a reason for less microleakage in restorations with preheated RCs [21]. It reconfirms that materials with lower viscosity tend to show a better surface penetration [23].

Other questions that rose during the performance of experiments have already been evaluated. One critical point seemed to be the pulpal warming as a result of the preheated dental RCs with temperatures up to 70 °C. Different *in vivo* and *in vitro* studies showed a low impact of the preheated RC, the thermal increase by means of the light curing process was identified to be higher [14,49,50]. Temperatures over 45 °C which may cause pain and physiological damage [29] or the pulpal tolerance describing a change of 10 °C have never been reached [51].

One of the biggest disadvantages in the daily clinical routine is that the preheated RCs loose temperature very quickly, up to 40%–50% after two minutes [52]. Consequently a preheated capsule (70 °C) will reach below mouth/body temperature after two to three minutes. That requires either a fast workflow or other additives, for example a heat storing applicator using metal on the front tip or even a heating device combined with the applicator. Another possibility could be a higher preheating temperature since until 140–200 °C no thermal activation is expected [14] and the pulpal warming is not yet an obstacle. Repeated or extended heating had no influence on the mechanical properties like degree of conversion [52] or *Knoop* hardness [53].

Comparing the SBS test with the contact angle measurements the positive trend in IFT values could not be observed with SBS results. IFT was calculated on the RC since the aim of the study was to evaluate changes in bonding characteristics of the RC to the ceramic. In contrast, the ceramic SBS specimens failed presumably in the ceramic substrate (cohesive fracture).

To conclude, *in vitro* investigations can never replace *in vivo* studies. However, different approaches give the possibility to minimize clinical failure. Based on the results of this study, the use of preheated RCs can be recommended.
